# Therapeutic potentials of *Crataegus azarolus* var. *eu- azarolus* Maire leaves and its isolated compounds

**DOI:** 10.1186/s12906-017-1729-9

**Published:** 2017-04-18

**Authors:** Eman Abu-Gharbieh, Naglaa Gamil Shehab

**Affiliations:** 1 Department of Basic Medical Sciences, College of Medicine, University of Sharjah, Sharjah, United Arab Emirates; 2 Department of Pharmacology and Toxicology, Dubai Pharmacy College, Dubai, United Arab Emirates; 3 Department of Pharmacognosy, Faculty of Pharmacy, Cairo University, Giza, Egypt

**Keywords:** *Crataegus azarolus* var. *eu- azarolus* Maire, 3β-O acetyl ursolic acid, Antimicrobial, Antihyperglycemic, Antihyperlipidemic

## Abstract

**Background:**

Hyperglycemia is a complicated condition accompanied with high incidence of infection and dyslipidemia. This study aimed to explore the phyto-constituents of *Crataegus azarolus* var. *eu- azarolus* Maire leaves, and to evaluate the therapeutic potentials particularly antimicrobial, antihyperglycemic and antihyperlipidemic of the extract and the isolated compound (3β-O-acetyl ursolic acid).

**Methods:**

Total phenolics and flavonoidal contents were measured by RP-HPLC analysis. Free radicals scavenging activity of different extraction solvents was tested in-vitro on DPPH free radicals. The antimicrobial activity of the ethanolic extract and its fractions as well as the isolated compounds were evaluated in-vitro on variable microorganisms. Animal models were used to evaluate the antihyperglycemic and antihyperlipidemic activities of the ethanolic extract along with the isolated compound (3β-O acetyl ursolic acid).

**Results:**

RP- HPLC analysis of the phenolics revealed high content of rutin, salicylic and ellagic acids. Six compounds belonging to triterpenes and phenolics were isolated from chloroform and *n*-butanol fractions namely: ursolic acid, 3β-O-acetyl ursolic acid, ellagic acid, quercetin 3-O-β methyl ether, rutin and apigenin7-O-rutinoside. Ethanolic extract showed the highest DPPH radical scavenger activity compared to other solvents. Ethanolic extract, hexane fraction, ursolic acid, 3β-O acetyl ursolic acid and quercetin 3-O-methyl ether showed variable antimicrobial activity against *E. coli, P. aeruginosa, S. aureus,* and *C. albicans*. Administration of the ethanolic extract or 3β-O acetyl ursolic acid orally to the mice reduced blood glucose significantly in a time- and dose-dependent manner. Ethanolic extract significantly reduced LDL-C, VLDL-C, TC and TG and increased HDL-C in rats. Ethanolic extract and 3β-O acetyl ursolic acid reduced in-vitro activity of pancreatic lipase.

**Conclusion:**

This study reveals that *Crataegus azarolus* var. *eu- azarolus* Maire has the efficiency to control hyperglycemia with its associated complications. This study is the first to evaluate antihyperglycemic and antihyperlipidemic potentials of 3β-O acetyl ursolic acid.

**Electronic supplementary material:**

The online version of this article (doi:10.1186/s12906-017-1729-9) contains supplementary material, which is available to authorized users.

## Background

Dyslipidemia and increased susceptibility to infections are two typical complications of diabetes mellitus. High glucose levels are highly associated with immune system impairment, particularly on neutrophils [1]. Hyperglycemia reduces the phagocytic activity and ability of neutrophils to form extracellular traps to kill bacteria [1]. On the other hand, hyperglycemia due to both insulin deficiency and insulin resistance significantly affect the lipid metabolic pathways [2]. Diabetic patients usually experience various cardiovascular complications of which dyslipidemia represents a main risk factor [2].

There has been a noticeable increase in the use of both traditional home remedies and herbal medicine instead of relying on conventional treatments [3]. This has caused traditional medicine to become of worldwide importance, with medicinal and economic effects [4].

*Crataegus azarolus* is indigenous to the Mediterranean Basin. *Crataegus azarolus* var. *eu- azarolus* Maire is a low, dense, spiny tree with a beautiful inflorescence up to 6 m tall and with orange fruits [5]. Phytochemical investigation of the plant was performed mainly on the flowers. Antioxidant activity and phenolic composition of the flowers extract were studied [6]. No available literatures concerning the pharmacological activity and the phytochemical constituents of the leaves were found.

In folk medicine, genus *Crataegus* (commonly called hawthorn in English and Zaarour in Arabic) is used for curing several ailments viz. central nervous, reproductive, cardiovascular and immune systems [7]. It also showed anti-inflammatory, cytotoxic, antioxidant, gastroprotective, antimicrobial, cardioprotective, antidiabetic and anti-HIV activities [8–14].

Phytochemical investigations on genus *Crataegus* were mainly performed on the leaves, flowers and berries. The isolated compounds were: oligomeric procyanidins, bioflavonoid, polysaccharides, catecholamines, vitamin C, saponins, tannins, cardiotonic amines, purine derivatives and ursolic acid [8–10, 15–18].

This study aimed to explore the phytochemical composition of *Crataegus azarolus* var. *eu- azarolus* Maire leaves’ extract, assess its acute toxicity and investigate the free radical scavenging and therapeutic potentials particularly antimicrobial, antihyperglycemic and antihyperlipidemic activities.

## Methods

### General

Shimadzu 1700 spectrophotometer was used for UV absorption spectra. Melting points were determined on Electrothermal 9100 equipment. Mass spectra were measured on a Jeol Mass Spectrometer SSQ 7000, Digital DEC 300. NMR spectra were measured in DMSO or CD_3_OD or CDCl_3_; ^1^H–NMR spectra were obtained at 400 MHz and ^13^ C-NMR spectra at 100 MHz on a JEOL GX-400 spectrometer with the chemical shifts (δ ppm) expressed relative to TMS as internal standard. Pre-coated silica gel 60 F_254_ (Merck, Darmstadt, Germany) was used for the TLC analysis. Vacuum liquid chromatography (VLC) was performed on silica gel 60 GF (Merck, Darmstadt, Germany). Sephadex LH-20 (Sigma-Aldrich, St. Louis, Missouri, United States) and silica gel 100 C_18_-Reversed Phase (Fluka, Switzerland) were also used. Analysis of phenolics was performed on Hewlett Packard HPLC (HP 1050HPLCDADw/Data System) equipped with a Hypersil-ODS (4.6 X 250 mm, 5 μm) column and a UV detector.

### Plant material

Leaves of *Crataegus azarolus* var. *eu- azarolus* were collected during the fruiting stage in July 2012 from plants cultivated in Nablus, Palestine. The plant was identified by Professor Hassnaa Ahmed Hosny, Department of Botany, Faculty of Science, Cairo University, Egypt. A voucher specimen has been kept at the Herbarium of the Department of Pharmacognosy, Faculty of Pharmacy, Cairo University.

### Chemicals, drugs and biochemical kits

All solvents were of analytical grade and obtained from Fisher Scientific. Sodium carboxymethylcellulose (CMC), 2,2-diphenyl-1-picrylhydrazyl (DPPH), gentamicin, fluconazole, alloxan, glibenclamide, cholesterol, lovastatin, orlistat, procaine pancreatic lipase type II, p-nitrophenyl butyrate (PNPB) and HMG-CoA reductase assay kit were purchased from Sigma-Aldrich (St. Louis, Missouri, United States). Folin-Ciocalteu reagent was obtained from Merck (Darmstadt, Germany). Lipid profile assessment kits including total cholesterol (TC), low density lipoprotein cholesterol (LDL-C), very low density lipoprotein cholesterol (VLDL-C), high density lipoprotein cholesterol (HDL-C) and triglycerides (TG) were purchased from Abcam-Cambridge, UK. Glucose estimation kit (kit Glu 1108, Test Strips, One Touch, Lifescan) was used to evaluate the blood glucose level.

### Plant leaves extraction

#### Selection of best extraction solvent for phenolic contents

Acetone, ethyl acetate, Methanol and 70% ethanol were used individually for the extraction of the powdered leaves material (each 100 g). Spectrophotometric methods were used to determine the phenolic and the flavonoid contents. The experiments were carried out in triplicate.

### Spectrophotometric determination of total phenolic and flavonoid contents

Folin-Ciocalteu reagent was used to measure the phenolic content according to Oktay et al. [19]. Different concentrations from gallic acid (10–50 µg/mL) were prepared for the standard calibration curve. The absorbance was determined at 750 nm. Results were calculated as mg gallic acid/g dry plant weight equivalent.

Aluminum chloride was used to assess the total flavonoid contents of the different extracts according to the procedure described by Dewanto et al. [20]. Serial dilutions of quercetin were used for preparation of the standard calibration curve. The absorbance was measured at 510 nm. All the experiments were carried out in triplicate.

### RP- HPLC analysis

Phenolic composition of *C. azarolus* var. *eu- azarolus* leaves was investigated in aliquots (1 g, each) of the methanolic extract of the plant via RP-HPLC [21, 22] based on the method previously described by the authors [23]. For the phenolic acids composition, the UV detector was set at 280 nm while for flavonoids composition the UV detector was adjusted at 330 nm. Quantification was based on peak area calculation and was done in triplicate.

### Large scale extraction and fractionation

Air-dried powdered leaves of *C. azarolus* var. *eu- azarolus* (2.5 Kg) were extracted at room temperature by maceration in ethanol (70%, 10 L × 3). The ethanolic extract was evaporated under reduced pressure at temperature 55 °C to provide 280 g residue. 200 g residue were successively fractionated with different solvents according to the polarity viz. *n*-hexane, chloroform and *n*-butanol saturated with water; while the remaining residual amount was saved for biological evaluation. Partitioning the ethanolic extract with different solvents yielded 20, 15 and 18 g of dried extractive respectively.

#### Isolation of the constituents of the chloroform extract

An accurately weighed amount of the chloroform extract (13.0 g) was applied on silica gel 60 GF VLC column (25 X 5 cm). Gradient elution was performed using hexane-chloroform, chloroform-ethyl acetate mixtures and ethyl acetate. Fractions (100 mL each) were gathered and monitored by TLC using different mobile phase (System A, chloroform-methanol 9.5:0.5 and System B, ethyl acetate: formic acid: acetic acid: water 10.0:1.0:1.0:0.5). Spots were located by visualization under UV_365_ nm before and after exposure to ammonia vapor and by spraying with *p*-anisaldehyde at 110 °C. Similar fractions were pooled to yield 6 collective fractions (F1-F6). According to the weight of the fraction and the number of spots, fractions F3 and F5 were selected for further isolation.

F3**:** (2.5 g; 6 spots, R*f* values 0.83, 0.70, 0.60, 0.50, 0.40 and 0.34, system A) upon rechromatography on a silica gel 100 C_18_-RP column under reduced pressure (20 X 1.5 cm), using methanol–water 9:1 as eluent, resulted in isolation of compounds 1 and 2.

F5**:** (1.2 g; 2 spots, R*f* values 0.77 and 0.47, System B) was exposed to three columns the first and second were on sephadex LH-20 (35 X 3 cm; elution, methanol–water 9:1 then 8:2) the third one was a silica gel 100 C_18_-RP under reduced pressure (20 X 1.5 cm; elution, methanol–water 1:1) to afford compound 3.

#### Isolation of the constituents of the n-butanol extract

The *n*-butanol residue (15 g) was fractionated by silica gel 60 GF VLC column (30 × 3.5 cm). Mixtures from different solvents were used (chloroform-ethyl acetate, ethyl acetate and ethyl acetate-methanol). Fractions (200 mL each) were gathered and seen by TLC (System B and System C, chloroform: methanol: water 8.5:1.5: 0.1). Spots were located before and after exposure to ammonia vapor by visualization under UV_365_ nm and by spraying with *p*-anisaldehyde at 110 °C. Similar fractions were pooled to yield 10 collective fractions (P1-P10). According to the weight of the fractions and the number of spots, fractions P7 and P8 were selected for further isolation.

P7: (1.5 g; 2 spots, R*f* values 0.72 and 0.50, System C) upon rechromatography on a silica gel 100 C_18_-RP column under reduced pressure(20 X 1.5 cm) using methanol: water 1:1 as eluent, resulted in isolation of compound 4.

P8: (0.8 g; 2 spots, R*f* values 0.38 and 0.22, System B) upon rechromatography on Sephadex LH-20 column (35 X 3 cm) using methanol: water 8:2 as eluent then on silica gel 100 C_18_-RP column (20 X 1.5 cm) using methanol: water 1:1 resulted in isolation of compounds 5 and 6.

### Antioxidant activity

#### DPPH radical scavenging assay

Ethanol (70%), methanol, acetone, and ethyl acetate were used individually to extract the leaves. The free radical-scavenging activity was assessed by DPPH radical [24]. Different concentrations of leaves extracts concentrations (3.9, 7.8, 15.6, 31.3, 62.5, 125, 250 μg/ml) and ascorbic acid (AA), as standard compound, were analyzed in triplicate. The percentage inhibition of DPPH radical was calculated as follow:

% inhibition = [A_0_ - (A_1_- A_2_)]/A_0_ ×100%.

A_0_: control absorbance, A_1:_ absorbance of the sample, A_2_: absorbance of the sample in ethanol without DPPH.

### Biological study

#### Microorganisms

Three bacterial strains and one fungus, were kindly provided by Rashid hospital (Dubai-UAE) and were used for the antimicrobial screening. This included one representative of the Gram-positive group (*Staphylococcus aureus* RMTCC 3161*)*, two representatives of the Gram-negative group (*Escherichia coli* RMTCC 2682 and *Pseudomonas aeruginosa* RMTCC 1687) and one fungus (*Candida albicans* RMTCC 5122)*.* Microorganisms were grown on appropriate media: nutrient agar for *S. aureus* and *P. aeruginosa,* MacConkey agar for *E. coli* and Sabouraud dextrose agar for *C. albicans.*

#### Antimicrobial activity

The ethanolic extract, and its fractions, *n*-hexane, chloroform and *n*-butanol as well as the isolated compounds of *C. azarolus* at doses of 375 μg/mL for each extract, and 140 μg/mL for the isolated compounds were subjected to in-vitro qualitative screening, for evaluation of their antimicrobial potentialities. The agar diffusion technique was used [25]. Solubilization of the samples was assisted by sterile DMSO. The effects were compared with gentamicin (30 μg/mL) and antifungal, fluconazole (30 μg/mL). Diameters of zones of inhibition (in mm) were taken as a measure for the growth inhibitory activity against the selected strains.

#### Experimental animals

Male albino mice (30 ± 5 g) were used for acute toxicity and antihyperglycemic studies. Antihyperlipidemic experiments were performed on Sprague Dawley rats weighing 210 ± 5 g. All animals were kept under standard conditions, fed with regular diet and water supplied ad libitum. Mice were accommodated for 1 week prior to the experiments. All animal investigations were accepted from the Ethical Research Committee of the Dubai Pharmacy College, Dubai UAE and done according to the ethical standards of laboratory animals [26].

#### Acute oral toxicity study

LD_50_ was determined by probit test [27]. Mice were divided into five groups (10 each) and they received various oral doses of the ethanolic extract (250, 500, 1000, 2500 and 5000 mg/kg). Later, they were observed over 72 h for any signs of morbidity or abnormal behavior and their death was recorded [27].

### Evaluation of antihyperglycemic activity

#### Induction of diabetes in mice

One night before the induction of hyperglycemia, the animals were kept fasted but given water ad libitum. The next morning, animals were injected 150 mg/kg alloxan monohydrate solution in acetate buffer (0.15 M, pH 4.5) intraperitoneally. The animals were observed over a week and their blood glucose values were measured. Mice with blood glucose levels between 180 and 360 mg/dL were assigned diabetic and were used later for further studies [28].

#### Oral glucose tolerance test

Animals were divided into four groups (*n* = 6 each). The mice were fasted for 18 h and provided water ad libitum. Each animal serves as its own control, Group I received only glucose at dose of 2 g/kg. Groups II and III received 250 and 500 mg/kg of the ethanolic leaves extract respectively, while group IV received the isolated compound in a dose of 50 mg/kg. All the tested samples were given orally 90 min before the glucose dose (2 g/kg, p.o.). Levels of blood glucose were calculated before and subsequently at 30, 60, 120 and 240 min after the administration of glucose dose. Blood glucose was measured by glucose estimation kit.

#### Experimental procedure

The diabetic animals were divided into five groups (*n* = 6 each). Group I kept as control, group II received 5 mg/kg glibenclamide as positive control, groups III and IV received the extract at doses of 250 and 500 mg/kg respectively, and group V received the isolated compound (2) at dose of 50 mg/kg. Levels of the blood glucose were measured pre and post (120 and 240 min) the treatment.

### Evaluation of antihyperlipidemic activity

#### Induction of hyperlipidemia in rats

The rats were fed daily by means of gavage tube with cholesterol at 25 mg/kg suspended in coconut oil given at 10 mL/kg daily for 30 days [29].

#### Experimental procedure

The animals were grouped into five treatment categories (*n* = 6, each), as following: Group I, kept as control group received daily 1% *w*/*v* sodium CMC suspension. Animals in groups II-V were hyperlipidemic and received daily cholesterol (25 mg/kg/day) in oil at 10:00 am. Group II represented the hyperlipidemic group, group III served as positive control and received lovastatin (10 mg/kg/day) at 3:00 pm. Similarly groups IV and V were given the ethanolic leaves extract at doses of 250 and 500 respectively at 3:00 pm. For a period of 30 days, the original and the final body weights and food intake of rats were monitored.

After this period, the fasted rats were sacrificed. Under ether anesthesia, samples of the blood were gathered by cardiac puncture. Lipid profile test was done including TC, HL-C, LDL-C, VLDL-C and TG.

Additionally, cardiac risk indicators were calculated by the “Atherogenic Index” TC: HDL-C ratio and LDL-C: HDL-C ratio.

#### In-vitro evaluation of the effect of the ethanolic extract and its isolated compound (2) on pancreatic lipase and HMGCoA reductase activities

In-vitro lipase inhibitory effect of the ethanolic leaves extract and the isolated compound (2) was assessed according to the method previously described [30]. The final concentrations of the tested samples of the plant extract and isolated compound were ranged from 50 to 500 μg/mL and 20–200 μg/mL respectively.

The following formula was used to calculate the percentage inhibitory activity (I):

$$ \mathrm{I}=100{\textstyle \hbox{-}}\left[\left(\mathrm{B}-\mathrm{b}\right)/\left(\mathrm{A}-\mathrm{a}\right)\times 100\right] $$

Where A: activity without inhibitor; a: negative control in absence of inhibitor; B: activity in presence of inhibitor; and b: negative control in presence of inhibitor.

Orlistat and DMSO were used as positive and negative control respectively and their activities were also tested.

For the in-vitro evaluation of HMG-CoA inhibitory activity, similar concentrations range of the plant extract and isolated compound were used. Pravastatin was used as standard drug with concentrations ranged from 0.1–2.5 μg/mL according to the method previously described [31]. HMG-CoA reductase inhibitory activity was calculated by using the following formula:

Inhibitory activity (I %) = (∆ Absorbance control- ∆ Absorbance test/∆ Absorbance control) × 100.

#### Statistical analysis

The results were expressed as mean + S.E.M (Standard Error Mean). Data was analyzed by GraphPad Software version 6.00 (San Diego, CA). One-Way ANOVA followed by Bonferroni’s multiple comparison tests against the control was performed. For repeated measures in glucose tolerance test, two-way ANOVA assessed the interactive and independent effects of treatment and time. *P* values < 0.05 were considered significant. IC_50_ values for the DPPH radical scavenging, pancreatic lipase Inhibition and HMG-CoA inhibition assays were determined from the dose–response curves using a linear regression analysis. For in-vitro evaluation of pancreatic lipase and HMG-CoA inhibition activities, inhibition of less than 40% was considered irrelevant and was selected as a cutoff point.

## Results

### Spectrophotometric determination of total phenolic and flavonoid contents

Different solvents were used for leaves extraction for the flavonoid and the phenolic contents to select the safest and the most effective extracting solvent as shown in Table 1. Spectrophotometric analysis revealed that ethanol was the best solvent to extract both flavonoids and phenolic acids.

**Table 1:** Flavonoid and phenolic acids contents of *C.azarolus* var. *eu- azarolus* leaves extracts

Solvent	Extraction yield (%)^a^	Total flavonoid content (mg quercetin/g)	Total phenolic content (mg GAE/g)
Ethanol	20.0 ± 1.2	1.5 ± 0.2	1.5 ± 0.6
Methanol	12.3 ± 1.7	1.1 ± 0.3	1.3 ± 0.7
Ethyl acetate	9.7 ± 0.8	0.9 ± 0.0	0.6 ± 0.0
Acetone	2.4 ± 0.3	0.7 ± 0.1	0.1 ± 0.0

^a^Expressed as 100 x (g dry extract/g dry leaves)

#### RP- HPLC analysis

RP-HPLC analyses of the methanolic leaves extract *of C. azarolus* var. *eu- azarolus* Maire revealed that 11 components were identified at *λ*=280 nm (corresponding to 37.73% of the total composition, Table 2) among which 8 were phenolic acids (30.77%) with prevalence of salicylic acid (11.91%) and ellagic acid (9.78%) and one flavonoid (catechin) besides the diphenol, catechol; meanwhile, at *λ*=330 nm, 7 components were known (Table 3); six of which were flavonoidal compounds with the major rutin (6.50%). (RP-HPLC chromatograms are available as Additional file 1).

**Table 2:** RP-HPLC analysis of phenolics components of *C. azarolus* var. *eu- azarolus* leaves extract at *λ* = 280 nm

Retention time	Identified component	Relative area %
6.81	Pyrogallol	0.17
6.92	Gallic acid	0.24
8.235	Protocatechuic acid	3.95
8.444	Catechin	4.82
8.593	Chlorogenic acid	2.97
8.950	Catechol	1.90
10.040	Caffeic acid	0.75
11.620	Ferulic acid	0.75
12.466	Salicylic acid	11.91
12.943	Ellagic acid	9.78
14.980	Cinnamic acid	0.47
Total identified constituents		37.73

**Table 3:** RP-HPLC analysis of phenolics components of *C. azarolus* var. *eu- azarolus* leaves extract at *λ* = 330 nm

Retention time	Identified component	Relative area %
3.83	Quercetin	0.01
11.78	Rosmarinic acid	0.89
12.44	Rutin	6.50
14.576	Narenginin	0.43
14.952	Hispertin	0.80
16.167	Apigenin	0.16
18.657	Chrysin	0.82
Total identified constituents		9.61

### Isolation of the constituents of the chloroform and n-butanol extracts

#### Isolated compounds

Compound 1 (ursolic acid): Molecular formula, C30 H48 O3; white powder; 20 mg; soluble in chloroform; Rf: 0.70 (System A); gave positive test for sterols and/or triterpenes; m.p., 286 °C; EI-MS m/z (% rel. Intensity) 456; ^1^HNMR (400 MHz, CDCl3): δH 0.79, 0.98, 0.97, 1.0 and 1.2 (15 H, 5 s, all CH_3_), 0.93, (3H, d, *J* = 6.4 Hz, H-30), 0.94 (3H, d, *J* = 6 Hz. H-29), 1.62 (2H, m, H-21), 1.38 (2H, m, H-16), 2.10 (d, 1H, *J* = 15 Hz, H-18), 3.32 (1H, dd, *J* = 10.8, 4.4 Hz, H-3), 5.30 (1 H, t; *J* = 3.6 Hz, H-12).

Compound 2 (3β-O acetyl ursolic acid): Molecular formula, C_32_ H_50_O_4_; white powder; 300 mg; soluble in chloroform; R_*f*_ *:* 0.60 (System A); gave positive test for sterols and/or triterpenes; m. p., 268–270 °C; EI-MS m/z (% rel. Intensity) 498; ^1^HNMR (400 MHz, DMSO): δ_H_ 0.76, 0.85, 0.87, 1.00, 1.1 (15 H, 5 s, CH_3_), 0.87 (3H, d, *J* = 6.4 Hz H-30), 0.91 (3H, d, *J* = 6 Hz. H-29), 1.21 (2H, m, H-21), 1.26 (2H, m, H-16), 2.15 (1H, d, *J* = 12 Hz, H- 18), 2.41 (3 H, s, OAc), 4.43 (1H, dd, *J* = 10.8, 4.4 Hz, H-3), 5.1(1 H, t, *J* = 3.6 Hz, H-12). ^13^C NMR data for compounds 1 and 2 were recorded in Table 4.

**Table 4:** ^13^C NMR (100 MHz) data of compounds 1 and 2

NO. C	Compound 1 (CDCl_3_)	Compound 2 (DMSO)
1	38.6	39.0
2	28.1	28.1
3	78.5	79.8
4	38.8	38.5
5	55.2	55.3
6	18.3	18.4
7	32.7	32.7
8	39.3	39.5
9	47.6	47.4
10	37.1	37.0
11	23.8	23.7
12	125.6	125.8
13	140.6	138.9
14	41.7	42.1
15	27.7	28.0
16	24.6	24.4
17	47.5	47.5
18	53.6	53.0
19	39.4	39.4
20	39.1	39.2
21	31.8	30.7
22	37.0	37.4
23	28.5	29.0
24	16.0	15.6
25	15.6	16.5
26	17.1	17.5
27	23.9	23.3
28	180.0	182.0
29	17.1	17.5
30	21.2	21.5
OAc		22.3
CH_3_COO		171.0

Compound 3 (ellagic acid): Molecular formula, C_14_H_6_O_8;_ yellow crystalline; 15 mg; soluble in methanol; R_*f*_ *:* 0.47 (System B); gave positive test for FeCl_3;_ m.p. 370 °C, EI-MS m/z (% rel. Intensity) 302; colour in visible light, yellowish brown; Under UV light and under UV/ammonia, brown, UV λ_max_ nm: CH_3_OH, 253, 354; ^1^H NMR (400 MHz, CD_3_OD): δ_H_ 7.49 (1H, s, H-5′) and 7.67 (1H, s, H-5).

Compound 4 (quercetin 3-O -β methyl ether): Molecular formula, C_16_H_12_O_7_, yellow powder, 35 mg; soluble in methanol; R_*f*_: 0.50 (System C); yellowish brown in visible light; brown under UV_365nm_ light and yellow under UV_365nm_/NH_3_, UV λ_max_ nm: CH_3_OH, 256, 294sh, 358; CH_3_ONa, 270, 331, 407; AlCl_3_, 274, 300sh, 336, 440; AlCl_3_/HCl, 264, 300sh, 360, 402; NaOAc, 273, 323, 380; NaOAc/H_3_BO_3_, 262, 297sh, 380; ^1^HNMR (400 MHz. DMSO): δ_H_ 3.84 (3H, s, OCH_3_), 6.21 (1H, d, *J* = 2 Hz, H-6), 6.49 (1H, d, *J* = 2 Hz, H-8), 6.84 (1H, d, *J* = 8.4 Hz, H-5′), 7.40 (1H, dd, *J* = 2.0, 8.0 Hz, H-6′), 7.70 (1H, d, *J* = 2 Hz, H-2′).

Compound 5 (rutin): Molecular formula C_27_H_30_O_16_; yellow powder; 20 mg; soluble in methanol; R_*f*_ *:* 0.38 (system B); m.p. 245 °C; brown in visible light, dull brown under UV_365nm_, and yellow under UV_365nm_/NH_3_; UV λ_max_ nm: CH_3_OH, 260, 266 sh, 300 sh, 356; CH_3_ONa, 278, 327, 411; AlCl_3_, 270, 301sh, 430; AlCl_3_/HCl, 267, 298, 360 sh, 400; NaOCOCH_3_, 269, 325, 390; NaOAc/H_3_BO_3_, 261, 300, 385; ^1^HNMR, (400 MHz, CD_3_OD): δ_H_ 6.11 (1H, d, *J* = 2 Hz H-6), 6.31 (1H, d, *J* = 2 Hz, H-8), 6.78 (1H, d, *J* = 8.4 Hz, H-5′), 7.57 (2H, d, *J* = 8.4 Hz,H-2′, H-6′); 3.33–3.67 (12H, m of sugar moieties), 4.41(1H, d, *J* = 1.5 Hz, Rham), 1.07 (3H, d, *J* = 6 Hz, CH-Rham), 5.2 (1H, d, *J* = 7.8 Hz, H-1 Glu).

Compound 6 (apigenin 7-O- β-D-glucopyranosyl (6 → 1)- α-L-rhamnopyranosyl-): yellow powder; 15 mg; soluble in methanol; R_*f*_ *:* 0.22 (system B); brown in visible light, dull brown under UV_365nm_, and yellowish green under UV_365nm_/NH3; UV λ_max_ nm: CH_3_OH, 267, 335; CH_3_ONa, 247 sh, 267, 300 sh, 385; AlCl_3_, 272, 300, 350, 381; AlCl_3_/HCl, 270, 300, 347, 380; NaOCOCH_3_, 254 sh, 265; 355, 385; NaOAc/H_3_BO_3_, 272, 373; ^1^H–NMR, (400 MHz, CD_3_OD): δ_H_ 7.91 (2H, d, *J* = 9.1 Hz, H-2′, H-6′), 7.03 (2H, d, *J* = 8.8 Hz, H-3′, H-5′)*,* 6.86 (1H, d, *J* = 2.2 Hz, H-8), 6.51 (1H, d, *J* = 2.2 Hz, H-6), 6.32 (1H, *s*, H-3), 3.14–3.53 (12 H, m, sugar moieties), 4.40 (1H, d, *J* = 2 Hz, −Rham), 1.07 (3H, d, *J* = 6.0 Hz, CH_3_-Rham), 5.1 (1H, d, *J* = 7.3 Hz, H-1 Glu). ^13^C NMR data for compounds 4–6 were recorded in Table 5.

**Table 5:** ^13^C NMR (100 MHz) data of compounds 4–6

NO. C	Compound 4 CD_3_OD	Compound 5 CD_3_OD	Compound 6 CD_3_OD
2	155.3	157.1	162.1
3	137.3	134.2	103.0
4	178.2	178.0	180.0
5	161.0	161.6	164.3
6	98.2	98.5	103.1
7	164.8	164.6	160.0
8	93.4	94.5	98.6
9	157.4	156.6	157.0
10	103.3	103.3	108.4
1’	121.3	122.2	122.4
2’	115.1	116.2	129.2
3’	144.7	145.5	116.4
4’	148.4	148.4	161.6
5’	115.2	116.1	116.4
6’	121.5	122.2	129.2
OCH_3_	58.2		
1”		101.2	103.3
2”		73.8	74.9
3”		76.8	77.3
4”		72.5	72.7
5”		75.8	76.7
6”		67.1	65.9
1″‘		102.3	102.8
2″‘		70.8	70.8
3″‘		70.0	71.2
4″‘		71.4	71.4
5″‘		69.1	69.1
6″‘		18.1	18.2

Compounds 1 and 2 gave positive Salkoweski reactions confirming their triterpenoidal nature [32]. ^1^H- and ^13^C–NMR spectra showed that compounds 1 and 2 were pentacyclic triterpene. Compound 1 was identified as ursolic acid while compound 2 was identified as 3β-O acetyl ursolic acid from their physical properties and their spectral data (^1^H–NMR and ^13^C–NMR) [33, 34].

The structure of compound 3 was identified as ellagic acid from its physical properties and different spectroscopic spectra (UV and^1^H–NMR) [35].

^1^H and ^13^C–NMR spectra of compound 4 demonstrated a methoxy group at δ_H_ 3.84 and at δ_C_ 58.2 respectively attached at position 3; compound 4 was identified as quercetin 3-O -β methyl ether [36].

The structure of compound 5 was identified as rutin from its physical properties and different spectral data (UV, ^1^H–NMR and ^13^C–NMR) [37].

**Fig. 1:**
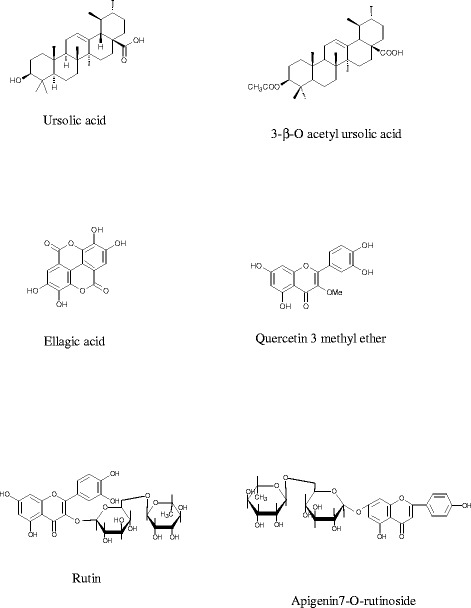
Compounds isolated from *C. azarolus* var*. eu- azarolus* leaves

The UV λ_max_ (335 nm) of compound 6 suggested that it possesses a flavone substituted skeleton. This was confirmed from the ^1^H–NMR spectrum. The occurrence of two doublets signals at δ_H_ 6.51 and 6.86 (*J* = 2.2 Hz) indicated the presence of two meta protons at C-6 and C-8 of ring A respectively. In addition, 2 doublets appeared at δ_H_ 7.03 (2H, *J* = 8.8 Hz,) and at δ_H_ 7.91 (2H, d, *J* = 9.1 Hz) indicated, H-3′, H-5’and H-2′, H-6′, respectively and one singlet at δ_H_ 6.32 for H-3. The presence of two anomeric signals at δ_H_ 4.40 (d, *J* = 2 Hz) and at 5.1 (d, *J* = 7.3 Hz) with the methyl group of rhamnose at δ_H_ 1.07 (d, *J* = 6.0 Hz) indicated the presence of 2 sugar units linked to C-7 position with 6 → 1 linkage. The sugar moieties were identified as glucose and rhamnose (TLC of acid hydrolysate, ^1^H–NMR and ^13^C spectral data). Compound 6 could be identified as apigenin 7-O- β-D-glucopyranosyl (6 → 1)- α-L-rhamnopyranosyl (apigenin 7-O-rutinoside) [38]. Figure 1 is showing the chemical structure of the isolated compounds.

### Antioxidant activity

#### DPPH free radical scavenging activity

**Fig. 2:**
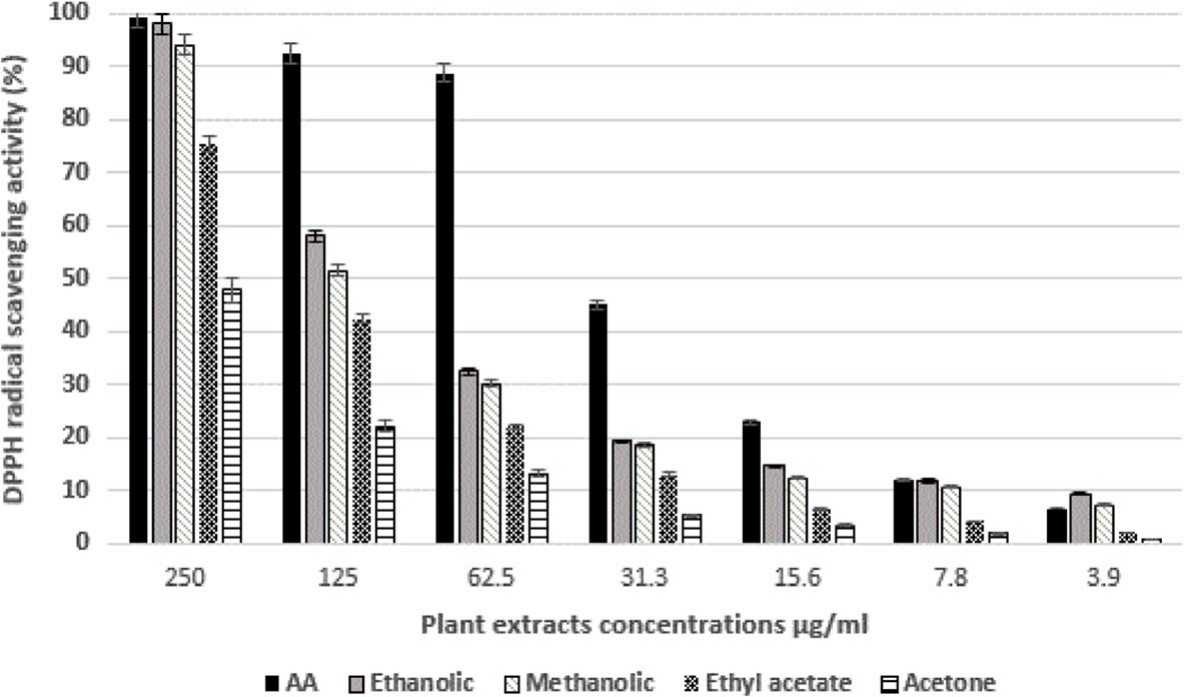
2,2-Diphenyl-1-picrylhydrazyl (DPPH) radical scavenging activity of different concentrations of different extracts of *C. azarolus* var. *eu- azarolus*. Ascorbic acid (AA) was used as positive control. The data presented as mean ± SD

DPPH free radical scavenging effects of the extracts were tested and the results are presented in Fig. 2. Both ethanolic and methanolic extracts showed the highest activity as revealed in Fig. 2. IC_50_’s of the leaves extracts were 129.2, 140.1, 164.1 and 262.3 μg/mL for the ethanolic, methanolic, ethyl acetate and acetone respectively. While for ascorbic acid, the IC_50_ was calculated to be 34.6 μg/mL.

### Biological studies

#### Antimicrobial activity

Results displayed in Table 6 revealed that at the tested concentrations, the ethanolic extract as well its *n*-hexane fraction and the isolated compounds, ursolic acid, 3β-O acetyl ursolic acid and quercetin 3-O- methyl ether, showed variable antimicrobial activity against all the tested pathogenic strains bacteria and fungus. On the other hand, all the tested samples exhibited variable antibacterial activities with inhibition zones ranging from 18 to 28 mm in diameter against *P. aeruginosa*.

**Table 6:** Antimicrobial activity of the ethanolic extract, fractions and isolated compounds of *C. azarolus* var. *eu- azarolus* leaves (zone of inhibition, mm)

Tested sample	*S. aureus*	*P. aeruginosa*	*E. coli*	*C. albicans*
Ethanolic extract	9.0 ± 1.1	22.0 ± 2.4	14.0 ± 1.2	5.0 ± 1.2
*n*-hexane	10.0 ± 1.8	24.0 ± 1.6	25.0 ± 2.6	7.0 ± 0.7
Chloroform	7.0 ± 0.7	25.0 ± 3.3	22.0 ± 3.0	-
*n*-butanol	-	20.0 ± 2.1	21.0 ± 2.1	-
Ursolic acid	23.0 ± 2.6	24.0 ± 2.4	22.0 ± 1.9	13.0 ± 1.2
3-β-O acetyl ursolic acid	30 ± 3.4	28.0 ± 3.1	29.0 ± 2.3	15.0 ± 0.8
Ellagic acid	-	23.0 ± 3.5	-	-
Quercetin 3-O- methyl Ether	25.0 ± 3.1	26.0 ± 2.9	25.0 ± 2.8	21.0 ± 2.3
Rutin	6.0 ± 2.5	20.0 ± 1.8	-	-
Apigenin7-O-rutinoside	6.0 ± 1.9	18.0 ± 1.6	-	-
Gentamicin	20.0 ± 2.7	18.0 ± 1.9	22.0 ± 3.4	-
Fluconazole	-	-	-	25 ± 2.5

The isolated compound 2, 3β-O acetyl ursolic acid, demonstrated the highest growth inhibitory activity against all the tested microorganisms, followed by quercetin 3-O- methyl ether and lastly, ursolic acid.

#### Acute oral toxicity study

The LD_50_ of the ethanolic extract of *C. azarolus* var. *eu- azarolus* Maire was safe up to 5000 mg/kg. During the observation period, no signs of morbidity or behavioral alteration in any animals’ groups were noticed.

#### Evaluation of antihyperglycemic activity

##### Oral glucose tolerance test

**Fig. 3:**
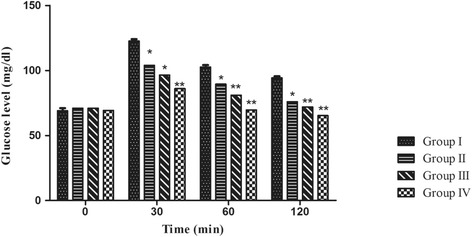
Effect of ethanolic extract of *C. azarolus* var. *eu- azarolus* leaves and 3β-O acetyl ursolic acid on glucose tolerance test, *p < 0.01, **p < 0.001. Group I received 2 g/kg glucose only. Groups II and III received 250 and 500 mg/kg of the plant extract respectively, and group IV received 3β-O acetyl ursolic acid

Blood glucose levels of normal mice were significantly reduced after receiving the plant extract at different doses in a time- and dose-dependent manner as shown in Fig. 3. Both doses of the leaves, as well as the isolated compound, 3β-O acetyl ursolic acid, exhibited significant antihyperglycemic effect. The effect was significant at 30, 120 and 240 min. Noticeable significant decrease in level of glucose was noticed at 30 min with the leaves extract at both doses as well as 3β-O acetyl ursolic acid at dose of 50 mg/kg (*p* < 0.01). This marked improvement in glucose tolerance was continued over the tested time.

##### Antihyperglycemic activity of the ethanolic extract and 3β-O acetyl ursolic acid on blood glucose levels in diabetic mice

The basal glycaemia was 241.7 ± 1.7 mg/dl for the diabetic control mice. There was no statistical difference between the glycaemic levels of the studied groups at time 0, (*p* > 0.05).

**Fig. 4:**
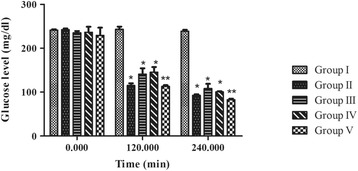
Effect of ethanolic extract of *C. azarolus* var. *eu- azarolus* leaves and 3β-O acetyl ursolic acid on glucose level in diabetic mice test, *p < 0.01, **p < 0.001. Group I is the control, group II: positive control and received glibenclamide, group III and IV were given the plant extract at doses of 250 and 500 mg/kg respectively, and group V was treated with the isolated compound

The anti-hyperglycemic activity of the ethanolic extract and the isolated compound (2) on the fasting blood sugar levels of diabetic mice is shown in Fig. 4. In diabetic mice, treatment of *C. azarolus* var. *eu- azarolus* leaves extract at dose of 250 and 500 mg/kg significantly lowered the basal level of blood glucose at 120 and 240 min (*p* < 0.01). A highly significant decrease of the blood glucose level was observed with 3β-O acetyl ursolic acid at the same timing intervals, *p* < 0.001.

#### Antihyperlipidemic activity

##### Body weight

Compared to cholesterol induced hyperlipidemic control group (group II), lovastatin treated group showed that the reference has no effect on the body weight (*p* > 0.05). On the other hand, groups received the ethanolic leaves extract showed a significant reduction in the percentage increment in body weight in day 15 and 30 (*p* < 0.01) as shown in Table 7.

**Table 7:** Percentage increment in the experimental groups’ body weight for 30 days treatment period

Group	Treatment (p.o.)	Day 0	Day 15	Day 30
I	1% w/v sodium CMC	211.3 ± 3.6	236.1 ± 4.3 (+11.9 ± 0.5)	253.2 ± 3.4 (+20.2 ± 0.4)
II	Cholesterol	209.3 ± 2.8	250.4 ± 3.6 (+19.8 ± 0.5)^#^	273.1 ± 4.6 (+30.5 ± 0.3)^#^
III	Cholesterol + lovastatin	212.4 ± 3.6	252.1 ± 2.4 (+18.7 ± 0.3)	272.1 ± 3.4 (+28.1 ± 0.6)
IV	Cholesterol + plant extract 250 mg/kg	211.8 ± 4.2	244.4 ± 5.1 (+15.4 ± 0.3)^*^	250.1 ± 3.5 (+18.1 ± 0.6)^**^
V	Cholesterol + plant extract 500 mg/kg	210.6 ± 5.3	241.1 ± 4.3 (+14.5 ± 0.2)^*^	247.8 ± 5.3 (+17.7 ± 0.8)\^**^

^#^ *p* < 0.01 vs group I; ^*, ﻿﻿**﻿﻿^ *p* < 0.05 and 0.01 vs cholesterol induced hyperlipidemic control group respectively

##### Effect of the ethanolic extract on serum lipid profile in hyperlipidemic rats

In hyperlipidemic model, groups treated with the ethanolic leaves extract and lovastatin showed significant reduction in TC, TG, LDL-C, and VLDL-C levels. In addition, serum HDL-C level was increased as compared to the control group (Table 8). Treated Groups with lovastatin and the leaves ethanolic extract demonstrated remarkable decrease in the “Atherogenic Index” and LDL-C: HDL-C risk ratios.

**Table 8:** Effect of *C. azarolus* var. *eu- azarolus* leaves extract on serum lipid profile, Atherogenic Index and LDL-C/HDL-C ratio in hyperlipidemic rats in hyperlipidemic rats (values are represented as Mean ± SEM)

Group	Total cholesterol (mg/dl)	Triglyceride (mg/dl)	HDL-C (mg/dl)	LDL-C (mg/dl)	VLDL-C (mg/dl)	Atherogenic index	LDL - C/HDL - C
I	61.7 ± 2.4	60.5 ± 5.6	36.3 ± 1.1	22.0 ± 0.7	12.4 ± 0.5	1.7 ± 0.3	0.6 ± 0.02
II	84.5 ± 2.5#	127.5 ± 2.5#	26.0 ± 1.1#	39.1 ± 1.3#	16.9 ± 0.6#	3.25 ± 0.7	1.5 ± 0.2
III	61.5 ± 0.5** (−27.2%)	58.0 ± 2.1** (−54.5%)	33.5 ± 0.5** (+28.8%)	24.3 ± 1.1** (−37.9%)	12.3 ± 0.1* (−27.2%)	1.8 ± 0.2	0.7 ± 0.05
IV	78.3 ± 3.4 (−7.3%)	61.3 ± 6.9** (−51.9%)	30.2 ± 1.4* (+16.2%)	26.5 ± 0.6** (−32.2%)	15.7 ± 0.7 (−7.1%)	2.6 ± 0.6	0.9 ± 0.04
V	61.7 ± 1.8** (−27.0%)	49.0 ± 2.5** (−61.6%)	32.5 ± 0.6** (+25.0%)	25.2 ± 0.7** (−35.6%)	12.9 ± 0.4* (−23.7%)	1.9 ± 0.3	0.8 ± 0.05

Group I: Control group received the vehicle, groups II-V were hyperlipidemic and received daily cholesterol (25 mg/kg/day) in oil at 10:00 am. Group II received cholesterol, group III received lovastatin, groups IV and V were given the plant extract at doses of 250 and 500 respectively. # *p* < 0.01 vs group I, * and ** *p* < 0.05 and 0.01 vs cholesterol induced hyperlipidemic control group respectively

##### In-vitro effect of the ethanolic extract and 3β-O acetyl ursolic acid on pancreatic lipase and HMGCoA reductase activities

*C. azarolus* var. *eu- azarolus* ethanolic extract at concentrations of 50–500 μg/mL reduced the activity of pancreatic lipase in-vitro. Significant reduction was noticed at concentrations of 200–500 μg/mL of 40.6–98.5% respectively with IC_50_ 252.3 μg/mL compared to orlistat (IC_50_ 0.59 μg/mL). Moreover, 3-β-O acetyl ursolic acid at concentrations between 80 and 200 μg/mL significantly reduced the pancreatic lipase activity with 42.4–99.6% and the IC_50_ was 93.6 μg/mL. On the other hand, the plant extract showed moderate inhibition of HMGCoA reductase activity at concentrations between 350 and 500 μg/mL of 42.6–60% with IC_50_ 394.1 μg/mL compared to pravastatin that has IC_50_ of 0.71 μg/mL. 3-β-O acetyl ursolic acid didn’t show any significant inhibition of HMGCoA at the tested concentration.

## Discussion

Diabetes Mellitus and other hyperglycemic disorders are complicated conditions associated with high prevalence of infection, dyslipidemia, hypertension and renal failure. The aim of this study was to find a standard plant extract that has the potential to control hyperglycemia with its associated complications, and to isolate and identify the active components that are responsible for those activities.

Results of total phenolic and flavonoid contents revealed that ethanol was the best solvent to extract both flavonoid and phenolic acids. Therefore, ethanol had been selected for further investigations. RP- HPLC analysis of the phenolics demonstrated high contents of rutin, salicylic and ellagic acids in the plant. Six compounds belonging to triterpenes and phenolic were isolated from chloroform and *n*-butanol fractions for the first time from *C. azarolus* var. *eu- azarolus* Maire. Ursolic acid is a triterpenoidal compound that finds in medicinal herbs, other plants and foods [39]. Ursolic acid showed anti-inflammatory, hepatoprotective, antihyperlipidemic, anticancer, inhibition of lipid peroxidation and antimicrobial activities [39–43]. Most of the available scientific papers are concerned about the activity of ursolic acid with no data regarding the antihyperglycemic and antihyperlipdimic of its acetate derivative. The most common approach to reversibly derivatize hydroxy groups is binding it with acyl groups. Thus, acetate prodrugs are characterized by favorable pharmacokinetic properties and rates of activation [44, 45]. Therefore, 3-β-O acetyl ursolic acid is assumed to have better pharmacokinetics features than ursolic acid itself. In-vitro DPPH assay was used to evaluate the free radical-scavenging effect of the leaves extracts of different solvents. The ethanolic extract showed the highest DPPH radical scavenger potential. This effect could be attributed to the ursolic acid and the phenolic compounds that were isolated from the ethanolic leaves extract. Ursolic acid, a pentacyclic triterpene, was reported to be a strong oxygen species (ROS) scavenger. Similarly, phenolic compounds including flavonoids particularly, quercetin 3-O -β methyl ether, rutin and apigenin 7-O- β-D-glucopyranosyl (6 → 1)- α-L-rhamnopyranosyl and phenolic acids (ellagic and salicylic acids) have been described to have high antioxidant effects [46–49]. Hyperglycemia worsens the development of infections and vice versa [50]. Blood glucose of more than 200 mg/dL has been strongly associated with reduced neutrophil activity [51]. Diabetic patients are at higher risk of infections from various microorganisms viz. *S. aureus*, *E. coli, P. aeruginosa* and *C. albicans* [52, 53]. The ethanolic leaves extract as well, its fractions and the isolated compounds exhibited noticeable antimicrobial activities against a wide range of microorganisms. This effect could support the immune system to fight against invading microorganism. Antipseudomonal activity of the chloroform extract could be ascribed to its isolated compounds namely, ursolic, 3β-O acetyl ursolic and ellagic acids through individual action or in a synergistic way. Other antimicrobial activities of chloroform fraction could be basically related to the triterpenes content only since ellagic acid appeared to be inactive on the other microorganisms. On the other hand, *n*- butanol fraction showed inhibition activity against both *P. aeruginosa and E. coli.* These activities are essentially linked to the phenolic constituents namely, quercetin 3-O-methyl ether, rutin and apigenin 7-O- β-D-glucopyranosyl (6 → 1)-α-L-rhamnopyranosyl. For the biological studies, doses were selected depending on the LD_50_ value (<1/10). Blood glucose levels of normal and diabetic mice received either ethanolic extract or 3β-O acetyl ursolic acid, were significantly decreased in a time- and dose-dependent manner. It has been previously reported that ursolic acid improves hepatic insulin resistance by stimulating the expression of peroxisome proliferator-activated receptors α (PPARα) [54]. Moreover, ursolic acid is reported to have high α-glucosidase inhibitory activity [55]. Those effects explained the antihyperglycemic activity of ursolic acid on fasting state and glucose tolerance test. Furthermore, ellagic acid is reported to possess antidiabetic action through inhibition of glycogen phosphorylase b enzyme [56]. Polyphenolic compounds, including quercetin 3-O -β methyl ether, rutin and apigenin 7-O- rutinoside are reported to have antihyperglycemic effect [57, 58]. Such combination of triterpenes and phenolic compounds could have synergistic antihyperglycemic actions. On the other hand, administration of the ethanolic leaves extract markedly decreased the percentile increment in body weight. Furthermore, the leaves extract significantly reduced the serum TC, TG, LDL-C, VLDL-C and increased HDL-C levels. Pancreatic lipase and HMGCoA reductase were used to explore the possible mechanisms for the antihyperlipidemic action. Both ethanolic extract and 3β-O acetyl ursolic acid reduced in-vitro activity of pancreatic lipase. In contrary, the extract showed moderate inhibition of HMGCoA reductase, while 3-β-O acetyl ursolic acid was unable to inhibit the enzyme activity at the tested doses. Based on that, antihyperlipidemic effect of the ethanolic extract can’t be solely contributed to HMGCoA inhibition. Thus, another mechanism could be suggested. Ursolic acid is reported to enhance the binding of PPAR-α to the response element in PPAR-α-responsive genes and modifies the lipid metabolism genes expression [59]. Thereby regulating the transcription of PPAR-α genes involved in lipid metabolism. Additionally, it reduces cellular cholesterol and triglyceride levels in hepatocytes, possibly by increasing the uptake and oxidation of fatty acid and by inhibiting their synthesis [59]. Interestingly, the enzymatic assay confirmed the inhibitory activity of the ethanoic extract and 3-β-O acetyl ursolic acid on pancreatic lipase. Hence, the antihyperlipidemic effect of the ethanolic extract and attenuation of body weight gain might be due to its inhibitory action on pancreatic lipase.

## Conclusion

The current study demonstrates the efficiency of the leaves extract of *C. azarolus* var. *eu-azarolus* Maire in controlling hyperglycemia with its associated complications such as infection and dyslipidemia. This multiple pharmacological profile might be due to the synergistic effect of its bioactive constituents including triterpenes, particularly ursolic acid and its acetyl derivative, and the phenolic compounds particularly, quercetin 3-O -β methyl ether, rutin and apigenin 7-O-rutinoside. This study is unique in the sense that it is the first to evaluate the antihyperglycemic and antihyperlipidemic potentialities of 3β-O acetyl ursolic acid.

## Additional file

Additional file 1: RP-HPLC chromatograms of the phenolic contents at *λ* =280 and 330 nm. (DOCX 566 kb)

## Abbreviations

DPPH: 2, 2-diphenyl-1-picrylhydrazyl
HDL-C: High density lipoprotein cholesterol
LDL-C: Low density lipoprotein cholesterol
PPAR: Peroxisome proliferator-activated receptors
TC: Total cholesterol
TG: Triglycerides
VLDL-C: Very low density lipoprotein cholesterol
