# Supplementary material for: Therapeutic potentials of Crataegus azarolus var. eu- azarolus Maire leaves and its isolated compounds
Source: BMC Complement Altern Med. 2017 Apr 18;17:218. doi: 10.1186/s12906-017-1729-9 (PMC5395866; doi:10.1186/s12906-017-1729-9)
Supplement: Additional file 1: — RP-HPLC chromatograms of the phenolic contents at λ =280 and 330 nm. (DOCX 566 kb) [file 12906_2017_1729_MOESM1_ESM.docx]

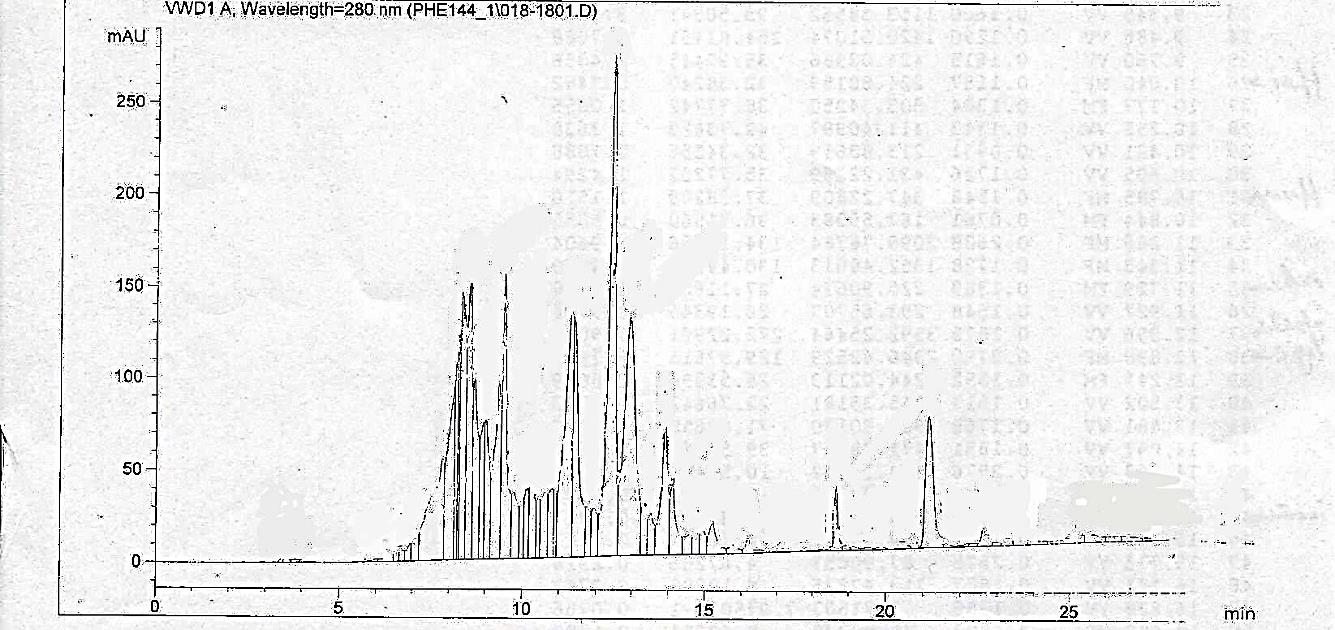


**Chart 1:** RP-HPLC chromatogram of the phenolic contents at 𝜆 =280 nm


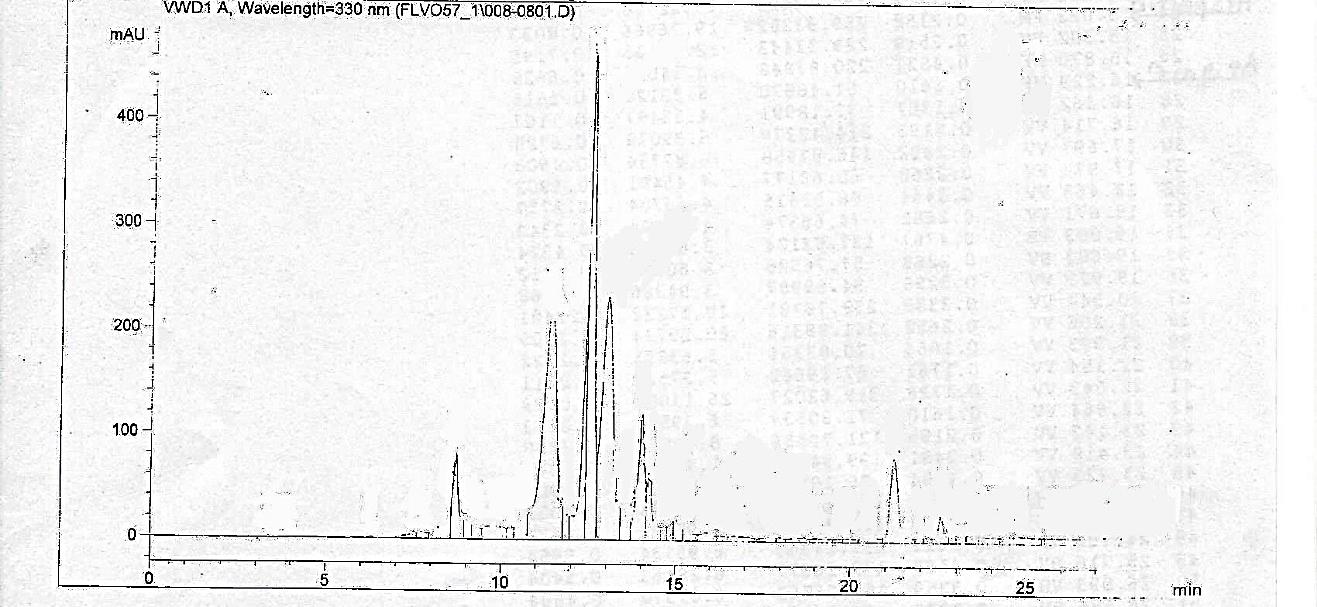


**Chart 2:** RP-HPLC chromatogram of the phenolic contents at 𝜆 =330 nm
